# Supplementary material for: A Paper-Based Electrochemical Sensor Based on PtNP/COFTFPB−DHzDS@rGO for Sensitive Detection of Furazolidone
Source: Biosensors (Basel). 2022 Oct 21;12(10):904. doi: 10.3390/bios12100904 (PMC9599777; doi:10.3390/bios12100904)
Supplement: Supplementary file 1 [file biosensors-12-00904-s001.zip › biosensors-1931782-supplementary.pdf]

Supporting Information

A paper-based electrochemical sensor based on PtNPs/$COF_{TFPB-DHzDS}$@rGO for sensitive determination of furazolidone

Rongfang Chen, Xia Peng, Yonghai Song and Yan Du*

National Engineering Research Center for Carbohydrate Synthesis/Key Lab of Fluorine, Silicon for Energy Materials and Chemistry of Ministry of Education, College of Chemistry and Chemical Engineering, Jiangxi Normal University, 99 Ziyang Avenue, Nanchang 330022, China.

* Correspondence: Tel: +86 0791 88120861. E-mail: wjxdu01@jxnu.edu.cn (Y.D.) or wjxdu01@163.com.

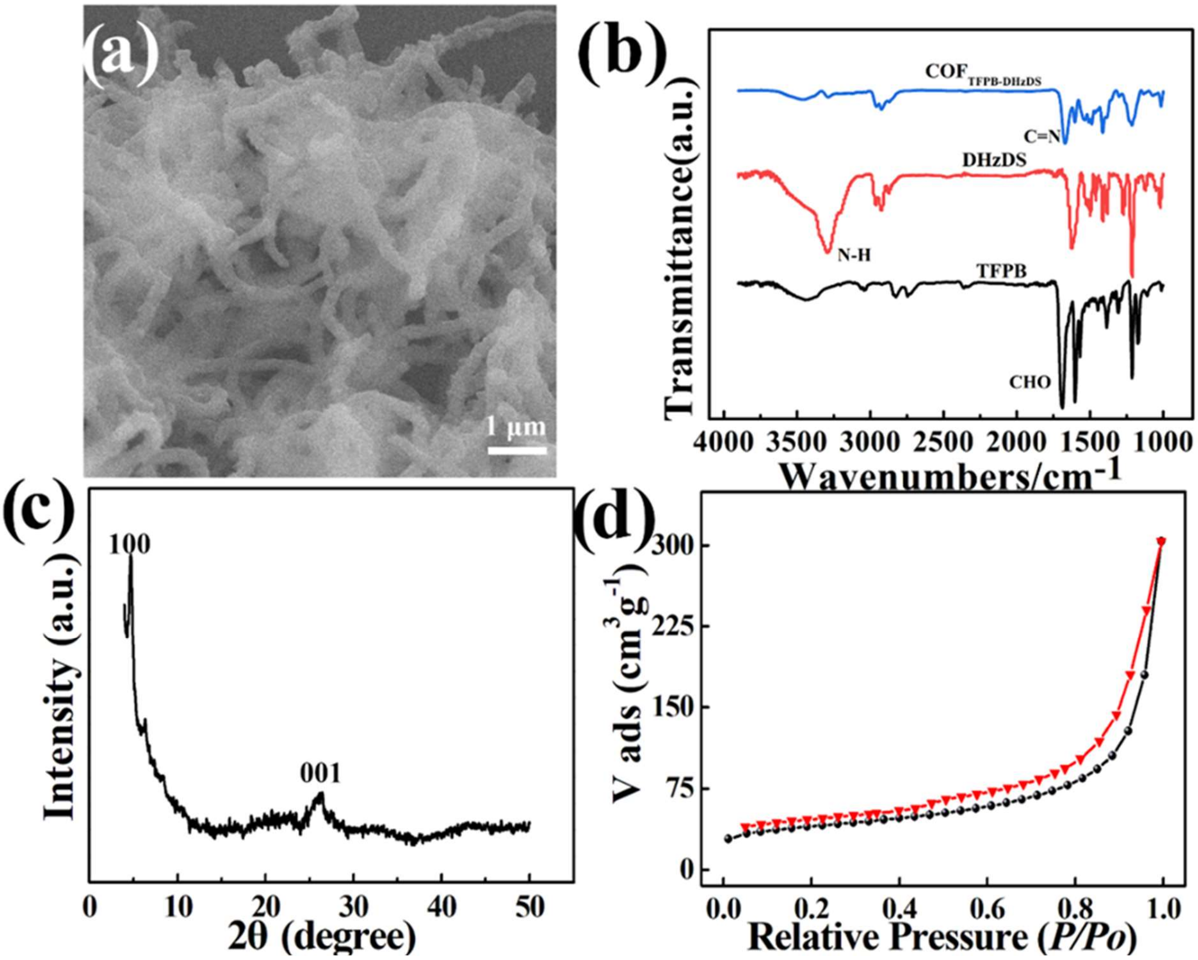

Figure S1. SEM image (a), FTIR spectrum (b), XRD pattern (c) and N_2 adsorption/desorption isotherm (d) of $COF_{TFPB-DHzDS}$.

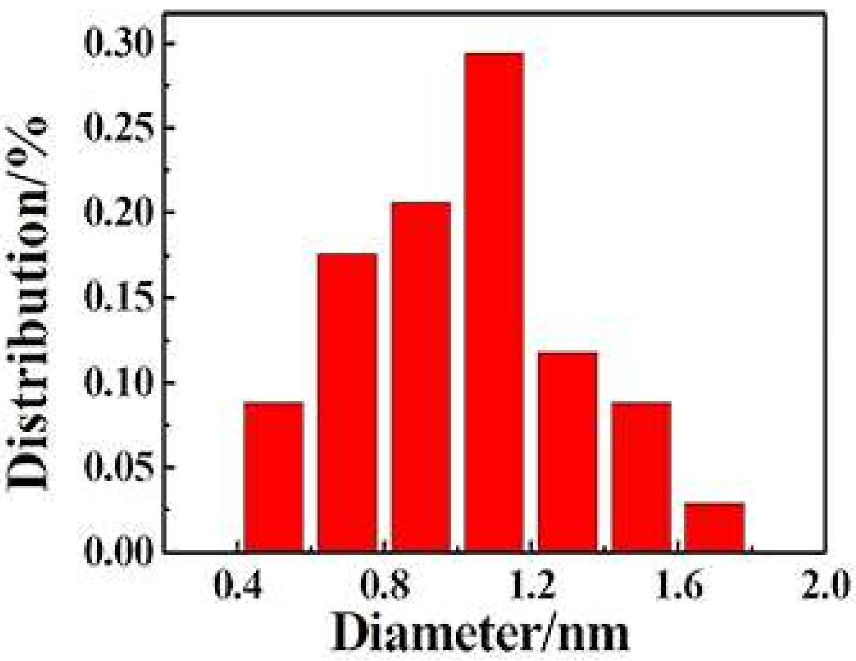

Figure S2. the particle size distribution of PtNPs in PtNPs/$COF_{TFPB-DHzDS}$@rGO.

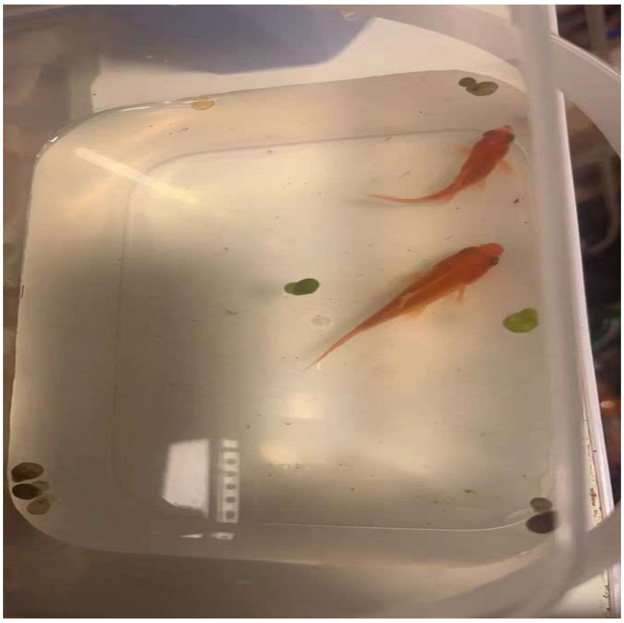

Figure S3. Picture of fish with furazolidone.

Table S1. The determination of furazolidone in human serum sample by PtNPs/$COF_{TFPB-DHzDS}$@ rGO/PBE.

Added (μM)	Found (μM)	Recovery (%)	RSD (%, n = 3)
0	-	-	-
20	19.5	97.5	2.5
40	40.2	100.5	0.5
60	59.6	99.3	0.6

Table S2. The determination of furazolidone in fish sample by PtNPs/$COF_{TFPB-DHzDS}$@rGO/PBE.

Added (μM)	Found (μM)	Recovery (%)	RSD (%, n = 3)
5	4.86	97.2	2.8
10	9.53	95.3	4.7
15	14.8	98.7	1.3
